# Benefits of Multimodal Exercise Intervention for BDNF and Cytokines Levels, Cognitive Function, and Motor Functionality in Alzheimer’s Disease: A Preliminary Study

**DOI:** 10.3390/ijerph22081245

**Published:** 2025-08-09

**Authors:** Emmanuel Dias de Sousa Lopes, Flávia Gomes de Melo Coelho, Sheilla Tribess, Jonatas da Silva Catarino, Bruno Naves Ferreira, Marina de Melo Reis, Antônio Ribeiro Neto, Carlo José Freire Oliveira, Jair Sindra Virtuoso Júnior

**Affiliations:** 1 Center of Research in Physical Activity and Health, Federal University of Triângulo Mineiro, Uberaba 38061-500, Minas Gerais, Brazil; emmanueldiaslopes@hotmail.com (E.D.d.S.L.); flaviaeduca@yahoo.com.br (F.G.d.M.C.); sheilla.tribess@uftm.edu.br (S.T.); ferreirabnef@gmail.com (B.N.F.); marina_meloreis@hotmail.com (M.d.M.R.); antoniorn11@yahoo.com.br (A.R.N.); 2 Laboratory of Immunology, Institute of Natural and Biological Sciences, Federal University of Triângulo Mineiro, Uberaba 38025-350, Minas Gerais, Brazil; jonatas.catarino@sf.mpg.de (J.d.S.C.); carlo.oliveira@uftm.edu.br (C.J.F.O.)

**Keywords:** Alzheimer’s disease, BDNF, physical exercise, elderly, cognition

## Abstract

Background: Physical exercise has been linked to improvements in motor and cognitive functions as well as to the modulation of neurotrophic and inflammatory factors, particularly in older adults. This aim of this study was to investigate the effects of a 12-week multimodal exercise program on cognitive function, motor performance, and plasma levels of brain-derived neurotrophic factor (BDNF) and cytokines in elderly individuals with Alzheimer’s disease (AD). Methods: A non-randomized controlled trial design was employed, involving 23 participants aged 62 to 85 years diagnosed with mild to moderate AD. The intervention group (*n* = 7) attended 60-minute sessions three times per week, incorporating aerobic, strength, flexibility, and motor coordination exercises, while the control group (*n* = 8) maintained usual activities. Methods: A non-randomized controlled trial design was used, involving 23 participants aged 62 to 85 years. Of these, 15 had a clinical diagnosis of mild to moderate AD and were allocated to either an intervention group (*n* = 7) or and AD control group (*n* = 8). The remaining eight participants were cognitively healthy and formed a control group matched for age and sex, used exclusively for baseline comparisons. The intervention group participated in 60-minute sessions three times per week, including aerobic, strength, flexibility, and motor coordination exercises. The AD control group (*n* = 8) maintained their usual daily routines. Results: Compared to baseline, the intervention group demonstrated significant improvements in executive and attentional functions, as measured by the Frontal Assessment Battery (FAB) and Clock Drawing Test (CDT); mobility, balance, gait speed, and lower limb strength also improved (*p* < 0.05). Additionally, plasma BDNF levels increased significantly, and interleukin-2 (IL-2) levels decreased. Conclusions: In conclusion, the multimodal exercise program resulted in cognitive and motor benefits and positively modulated biomarkers related to neuroplasticity and inflammation, supporting its potential as a complementary intervention in elderly individuals with AD.

## 1. Introduction

The number of elderly people with Alzheimer’s disease (AD) is growing due to increasing life expectancy; however, other contributing factors such as medication use, comorbid conditions, lifestyle habits, and ethnicity should also be considered [1,2]. According to Alzheimer’s Disease International (ADI), there are an estimated 46.8 million people in the world with AD [3]. The effect of pharmacological treatment is limited, and there is an urgent need for alternative therapeutic treatments. In this sense, non-pharmacological approaches, such as occupational therapy, cognitive stimulation, training of caregivers, psychotherapy, social activities and physical exercise, have been employed with the aim of attenuating the symptoms of the disease [4,5].

Physical exercise has gained prominence in the scientific literature as a non-pharmacological treatment for AD in the elderly due to its effectiveness in promoting improved cognitive function, including executive function, attention, and language [6,7,8], and motor function, including gait [7], postural control, muscle strength [6,9], balance, and mobility [8,10]. In the studies of AD, one systematic review suggested that physical exercise could lead to significant improvement in functional capacity components (flexibility, agility, balance, and strength), cardiovascular fitness, and performance of daily life activities; improvement in depressive symptoms and neuropsychiatric disturbances and improvement in some cognitive components such as sustained attention, executive function, and visual memory in elderly patients with AD. Regarding the type of exercise, multimodal protocols were the most widely applied and have shown positive outcomes [5]. It is probably even more effective to combine complex physical training with cognitive training [11]. Regarding the parameters related to the intensity and duration of exercise, beneficial effects were predominantly derived from exercises performed at a moderate intensity for at least 30 min per session [5].

Although the literature highlights the benefits of physical activity in older adults with AD, it is important to recognize that different exercise modalities may exert distinct effects on motor and cognitive domains. Group-based interventions that emphasize motor coordination, postural balance, and social interaction, such as dance, have shown promising results [12]. These modalities, by integrating motor, sensory, cognitive, and social stimuli, appear to act synergistically in promoting neuroplastic processes, with positive effects on motor and cognitive performance [12,13]. In the study by Bianco et al. [13], for example, older adults participating in structured dance programs, such as waltz, mazurka, and polka, demonstrated improvements in functional balance and a reduction in fall risk. Although these findings are not specific to individuals with AD, they provide additional support for the adoption of multimodal approaches with the potential to preserve and enhance functionality in older adults.

Although the literature highlights the positive effects of physical exercise on cognitive function in older adults, there is still a scarcity of longitudinal studies investigating the impact of exercise on the synthesis of neurotrophic factors, particularly brain-derived neurotrophic factor (BDNF), which is relevant for neuroplasticity processes and the maintenance of cognitive function. Gholami et al. [14] conducted a systematic review with meta-analysis and found that older adults who participated in physical training programs lasting more than four weeks showed increased circulating BDNF concentrations, with a particular emphasis on aerobic exercises. The intensity of these interventions was predominantly moderate, often measured using perceived exertion levels ranging from 12 to 14 on the Borg scale. Erickson et al. [15], in a randomized clinical trial involving 120 older adults who underwent aerobic treadmill training for one year (three times per week at 60–75% of heart rate reserve), observed increased serum BDNF levels, which were associated with greater hippocampal volume and improved memory. In patients diagnosed with AD, increases in BDNF levels have also been observed following moderate-intensity aerobic exercise interventions, typically involving 30- to 45-minute sessions three to five times per week, with perceived exertion levels between 11 and 13 on the Borg scale. The present study aimed to analyze the effect of a multimodal exercise intervention on BDNF levels (primary outcome) as well as on cognitive function (attention, executive function, and language) and motor function (balance, gait, and muscle strength) in older adults with AD, compared to matched controls.

## 2. Materials and Methods

### 2.1. Participants

This was a non-randomized controlled trial with pre–post measurements study that initially included 50 elderly patients with AD. All patients were community-dwelling citizens in Uberaba, Minas Gerais, Brazil, and all had a diagnosis of AD according to a neurologist. Sample selection was carried out through both active and passive recruitment strategies. Active recruitment was conducted by the principal investigator through visits to neurology and geriatrics clinics and contact with primary healthcare professionals. Passive recruitment included dissemination of the project through local media (radio, newspaper, and television) and partner institutions such as the ABRAz (Associação Brasileira de Alzheimer—Brazilian Alzheimer’s Association). Participants in the intervention group were exclusively selected from those who voluntarily enrolled in the extension project “MoviMente—Physical Exercise for Older Adults with Alzheimer’s Disease”, affiliated with the Federal University of Triângulo Mineiro. The control group consisted of individuals recruited from other locations and institutions not associated with the intervention, in order to minimize the risk of contamination between groups and ensure the independence of exposures. Matching between groups was performed based on age, sex, and degree of cognitive impairment.

The sample size was estimated using G*Power software (version 3.1.9.2), considering a repeated-measures ANOVA (within-between interaction), with a statistical power of 90%, a significance level of 0.05, and a moderate effect size (f = 0.25). The analysis indicated a minimum requirement of 35 participants. To reach this number, 50 older adults were initially assessed for eligibility, anticipating a dropout or exclusion rate of up to 25%. However, after applying the inclusion and exclusion criteria, only 23 participants met the eligibility requirements and were included in the study, while 27 individuals were excluded. These participants were distributed across three groups: an exercise group with AD (*n* = 7), an AD control group (*n* = 8), and a cognitively healthy control group (*n* = 8), matched for age and sex. These participants were distributed across three groups (7 + 8 + 8 = 23), and the cognitively healthy control group was used solely for baseline comparisons of BDNF, IL-2, and cognitive variables. Motor function outcomes were not assessed in this group.

This reduced sample size resulted in an estimated actual statistical power of approximately 80%.

Regarding blinding, although participants could not be blinded to their intervention status, all cognitive and functional assessments were conducted by previously trained assessors who were blinded to participants’ group allocation. The use of blinded assessors aimed to reduce measurement bias and enhance the internal validity of the results obtained.

The following inclusion criteria were adopted for participation in the study: elderly patient with a clinical diagnosis of AD, according to the Diagnostic and Statistical Manual of Mental Disorders [16], and mild or moderate dementia according to the Clinical Dementia Rating Score (CDR) [17]. In addition, patients were required to be available for participation during the evaluations proposed by the researcher.

**Figure 1:**
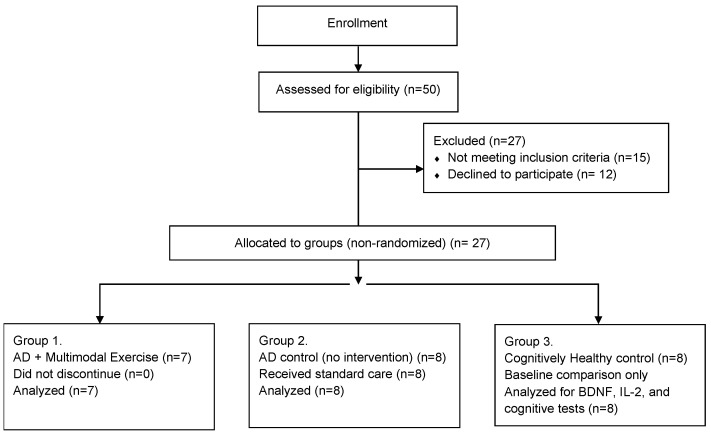
Flowchart of Participant Inclusion and Group Allocation (Non-Randomized Design), *n* = 21.

Figure 1 shows the sample recruitment as well as the reasons for non-inclusion of participants in the study. Although the procedure was not random, it is important to mention that the groups did not differ in any of the variables at baseline.

The baseline characteristics of participants included in the three groups were as follows:

Intervention group with AD (*n* = 7): age between 62 and 85 years (mean = 75.8; SD = 6.7), 3 three men and four women, average education of 7.6 years (SD = 4.9), mean CDR score of 1.5 (SD = 0.5), mean depressive symptoms score of 7.5 points (SD = 2.7), mean MMSE score of 18.6 (SD = 4.7), and mean QBMI score of 1.8 points (SD = 1.3).

Control group with AD (*n* = 8): age between 63 and 89 years (mean = 76.1; SD = 7.9), one man and seven women, average education of 8.6 years (SD = 5.7), mean CDR score of 1.6 (SD = 0.7), mean depressive symptoms score of 6.9 points (SD = 2.3), mean MMSE score of 17.0 (SD = 7.3), and mean QBMI score of 2.9 points (SD = 1.7).

Control group without AD (*n* = 8): age between 62 and 81 years (mean = 75.3; SD = 7.7), two men and six women, average education of 7.6 years (SD = 1.9), CDR score equal to 0 (no cognitive impairment), mean depressive symptoms score of 3.2 points (SD = 1.6), mean MMSE score of 28.8 (SD = 1.4), and mean QBMI score of 3.5 points (SD = 0.9).

All groups were included in the statistical analyses presented throughout the manuscript. However, the clinical and sociodemographic data of the control group without AD are not included in Table 1 and are presented in the text only, in a descriptive manner.

### 2.2. Procedures

Participants were evaluated at baseline and after 12 weeks. The training group participated in a multimodal exercise intervention. The control group kept to their same daily routine and did not participate in any regular or structured exercise programs. All participants, independent of group, continued to take their regularly prescribed medications.

A structured interview was conducted by the researcher to obtain socio demographic data, such as age, sex, and educational level, and clinical characteristics, including disease duration, medications taken (name and daily doses) and general comorbidities. The classification of the degree of impairment was made according to the CDR [17], which evaluates cognition and behavior and takes into account the influence of cognitive losses on the patient’s ability to adequately perform daily life activities, classifying the degree of dementia as 0 (no changes); 0.5 (questionable dementia); 1 (mild dementia); 2 (moderate dementia); and 3 (severe dementia). Level of physical activity was assessed using the Modified Baecke Questionnaire for the Elderly, which evaluates the level of physical activity in tasks performed at home, sports, and leisure activities [18,19]. Additionally, the Modified Baecke Questionnaire was also administered post intervention in the control group to monitor potential changes in physical activity levels throughout the study period. The Mini-Mental State Examination (MMSE) [20] was used for the global cognitive evaluation. Depressive symptoms are known to interfere with cognitive in the elderly. For the identification of possible interference, the Geriatric Depression Scale (GDS) was used [21].

### 2.3. BDNF Plasma Concentration

Baseline BDNF was measured in a blood sample taken after a 30-minute rest period seated in a chair, immediately prior to the exercise test (pre). At the end of the exercise, a second blood sample was drawn for BDNF analysis (post). These samples were collected before and after 12 weeks of the experiment (exercise or control). Blood was collected into tubes with EDTA anticoagulant. The tubes were placed on ice and then centrifuged for 30 min at 3000× *g* rpm at 8 °C. The plasma was collected and frozen at −80 °C until further analysis. BDNF levels were measured by Enzyme-Linked Immunosorbent Assay using the BDNF Emax^®^ ImmunoAssay System (Promega, Madison, WI, USA) according to the manufacturer’s instructions. All samples and standards were measured in duplicate, and the means of duplicates were used for statistical analyses.

To control for circadian variations in BDNF levels, all blood samples (pre- and post-intervention) were collected between 7:00 and 9:00 a.m. [22], at rest, and not after an acute bout of exercise. This procedure was standardized for all groups and time points to avoid confounding acute responses with baseline levels.

### 2.4. Cytokine Assays

The quantification of TNF-α, IFN-γ, IL-2, IL-4 e, and IL-10 was assessed in plasma using the Cytometric Bead Array (CBA) (BD Bioscience, San Jose, CA, USA), following the manufacturer’s instructions.

### 2.5. Cognitive Function Evaluation

The Frontal Assessment Battery (FAB) [23] was used to evaluate executive function and attention and is composed of 6 subtests: “Similarities” (abstract reasoning), “Lexical Fluency” (mental flexibility), “Motor Series” (programming), “Conflicting Instructions” (sensitivity to interference), “Go—do not go” (inhibitory control), and “Holding Behavior” (primitive reflex). The test scores range from 0 to 18 points, and higher scores indicate better performance in frontal functions. The Clock Drawing Test (CDT) [24] was also utilized to measure executive functions such as planning, abstract thinking, logical sequencing, and monitoring of executive processing. The test is scored from 0 to 10 points, and higher scores indicate better performance in the executive functions evaluated. To evaluate language, the Semantic Verbal Fluency Test (SVFT) [25] was utilized. The assessment is quantified by the ability to name as many animals as possible in one minute.

### 2.6. Motor Functionality

Motor function was evaluated using the Brazilian Version of the Short Physical Performance Battery (SPPB) [26], which is composed of three tests to evaluate balance, gait speed, and lower limb strength. Each of the tests is given a score of 0 to 4 points, constituting a total score of 0 of to 12 points. The Time Up and Go test [27] was used to assess dynamic balance and agility and the Berg Functional Balance Scale [28] to measure functional balance.

### 2.7. Intervention

Three weekly multimodal exercise sessions of 60 min duration were conducted and supervised by an experienced physical education professional for a period of 12 weeks. The intervention lasted 12 weeks, consisting of 6 weeks of motor training followed by 6 weeks of combined cognitive–motor tasks. The protocol was based on the recommendations of the American College of Sports Medicine [29], which suggests that the practice of physical exercise for the elderly should include aerobics, flexibility, balance, and muscle strengthening exercises. Aerobic capacity and flexibility were components worked in all classes. During the first six weeks of the intervention (1st to 6th weeks), the elderly patients were instructed to perform coordination, aerobic resistance, strength, flexibility, balance, and agility exercises to promote motor development and aerobic capacity. Activities including stretching, weight training, circuits, pre-sports games, dance sequences, playing activities, and relaxation were prescribed. Auxiliary materials were used, such as dumbbells, anklets, sticks, gymnastics balls, cones, arches, steps, and mats. In the last six weeks of the intervention (7th to 12th weeks), dual-task activities were initiated. The motor training from the first six weeks was associated with cognitive tasks (for example, countdown; recognition of shapes, colors, animals, fruits, and objects; tasks of verbal fluency, etc.) to verify if the elderly patient was able to carry out a motor activity simultaneously with a frontal cognitive activity that requires concentrated attention, planned organization of answers, abstraction, judgement, and mental flexibility as well as to search for semantic meaning in word generation. During the intervention, the degree of cognitive and motor complexity was increased. The increase in the actions of cognition stimuli was applied as the tasks became easier for the elderly patients to add increased complexity to the tasks. For example, there was an increase in the number of figures named while performing the movement of stepping in and out of step. The heart rates of participants were assessed using a frequency meter (model A4; PolarElectro O) during the sessions. Blood pressure was also measured during the sessions to ensure that the training respected the individual conditions of the participants [30].

In weight training, progression occurred every two weeks, as described in Table 2.

The intensity of aerobic training was maintained between 65% and 75% of the predicted maximum heart rate for age (220—age) during the 12 weeks, characterizing a training with aerobic predominance of moderate intensity [29]. The control group was instructed to continue with their usual daily activities. 

### 2.8. Data Analysis

The sociodemographic and clinical are presented as mean and standard deviation. The Shapiro–Wilk test was used to assess the normality of the data. Sociodemographic and clinical characteristics at baseline were compared between groups using the Mann–Whitney U-test. Additionally, effect sizes (r) were calculated for the non-parametric tests (Mann–Whitney U and Wilcoxon) to assess the magnitude and practical significance of the observed differences beyond *p*-values. These values are presented in the footnotes of Table 2. Given the small sample size and exploratory nature of this pilot study, no correction for multiple comparisons was applied. BDNF was considered the primary outcome. Other outcomes (IL-2, cognitive, and motor variables) were treated as exploratory.

The differences between groups in outcomes (BDNF levels, cognition functions, and motor functionality) at baseline and follow-up at 12 weeks were analyzed using the Mann–Whitney U-test. The differences in outcomes from baseline to follow-up at 12 weeks for both groups were analyzed using the Wilcoxon test. A significant level of 5% (*p* < 0.05) was adopted for all analyses. Data analysis was performed using SPSS software, version 21.0. Given the exploratory nature and small sample size of this preliminary study, no correction for multiple comparisons was applied to avoid inflation of type II error. However, effect sizes (r) were calculated and reported to support interpretation of practical significance.

## 3. Results

The results refer to the three study groups: the intervention group with AD (*n* = 7), the control group with AD (*n* = 8), and the control group without AD (*n* = 8), totaling 23 participants. All groups were included in the statistical analyses for both primary and secondary outcomes. Clinical and sociodemographic comparisons between the groups with AD (intervention and control) are presented in Table 1. The descriptive characteristics of the control group without AD are provided exclusively in the text (Section 2.1). No statistically significant differences were observed between the intervention group and the control group with AD in any of the clinical and demographic parameters evaluated (Table 1).

**Figure 2:**
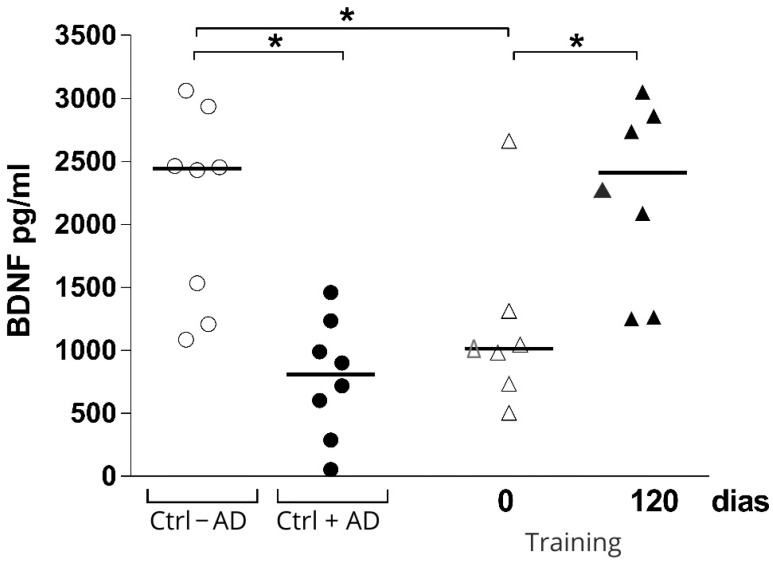
Brain-derived neurotrophic factor plasma concentration at baseline and after 12 weeks in the intervention group (Training), control group without AD (Ctrl − AD), and control group with AD (Ctrl + AD). * *p* = 0.03.

Regarding BDNF levels, the Mann–Whitney U-test showed differences between groups without AD compared with AD baseline. The Wilcoxon test detected significant increases in BDNF levels from baseline to follow-up at 12 weeks in the intervention group; surprisingly, the levels of BDNF in the intervention group were similar to the control group without AD (Figure 2).

The Mann–Whitney U-test did not show differences between groups at baseline and follow-up at 12 weeks in cognitive function. However, after 12 weeks of intervention, benefits were observed in cognitive function in the intervention group.

The Wilcoxon test detected higher scores in the FAB and in the CDT of the intervention group, but no significant differences were found for the SVFT test. In order to assess the efficacy of the intervention, comparisons were specifically performed between the intervention group and the control group with AD. Although between-group differences did not reach statistical significance in the Mann–Whitney U-test, the within-group improvements observed in the intervention group for FAB and CDT strengthen the interpretation of a beneficial effect. For motor variables, the test did not show differences between groups at baseline and follow-up at 12 weeks (Table 3). The Wilcoxon test detected higher scores in the SPPB of the training group. In addition, the training group had reduced time during the execution of the gait speed and in the execution of the standing up test for muscle strength. In the Timed Up and Go Test (TUG) test after the 12-week program, the participants reduced their time during the execution of the test, although there was no reduction in the number of steps. There was no significant difference between the groups on the Berg Functional Balance Scale.

**Figure 3:**
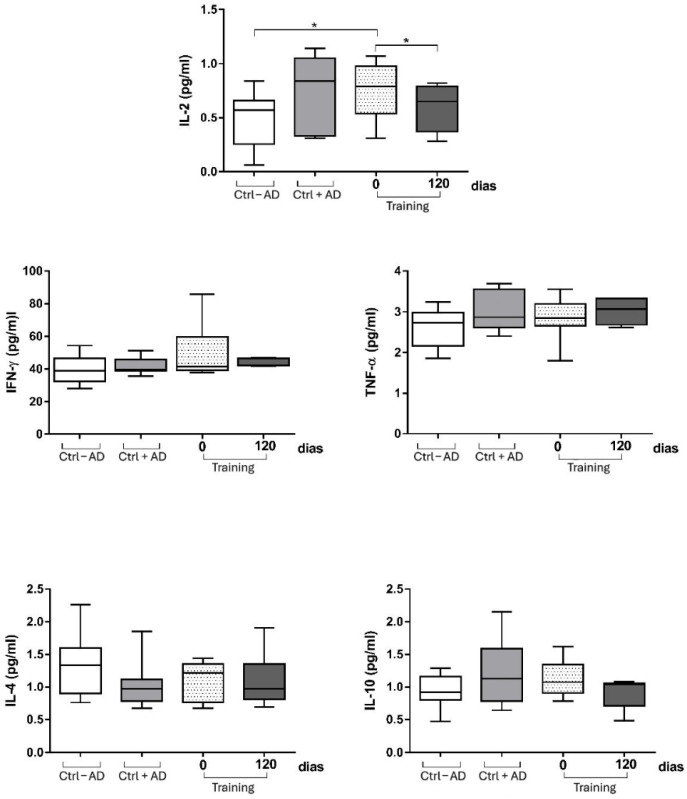
Levels of cytokines among the elderly with or without Alzheimer’s disease at baseline and after 12 weeks in the intervention group (Training), control group without AD (Ctrl − AD), and control group with AD (Ctrl + AD). * *p* < 0.05 compared with baseline within the same group (Wilcoxon test).

When we analyzed the production of cytokines among the elderly with or without Alzheimer’s disease, we observed that the elderly with Alzheimer’s have increased IL-2 production. When the Alzheimer’s patient group was submitted to the physical activity protocol, there was a significant decrease in serum IL-2 levels in these individuals. The decrease in this group was so significant that the levels of this cytokine were like the levels of the elderly group without the disease (Figure 3). When we analyzed cytokines associated with the Th1 (INF-γ, TNF-α), and Th2 (IL-4) and the T regulator (IL-10) immune response profiles, we demonstrated that multimodal physical training did not influence the levels of these cytokines at time 0 (pre-exercise levels) and 120 days after intervention (Figure 3).

## 4. Discussion

The results of the present study suggest that for the sample of elderly patients with AD who performed 12 weeks of a specific multimodal exercise intervention, plasma BDNF levels increased. In addition, we found a possible effect on cognition and motor functionality in subjects adhering to the program. There are still few studies that longitudinally assess the effects of a multimodal physical exercise protocol on peripheral BDNF concentrations in elderly individuals with AD. Studies have shown that chronic physical exercise increases peripheral levels of BDNF (plasma or serum) of healthy older adults [14,31] as well as individuals with neuropsychiatric and metabolic diseases, such as mild cognitive impairment (MCI) and diabetes [32].

With regard to the type of physical exercise, aerobic training, resistance training, and multimodal physical exercise have all been used in studies to evaluate the effect of exercise on BDNF levels in the elderly. Elderly patients without dementia who participated in moderate aerobic training on the treadmill over six months had increased BDNF levels [14]. Another study showed that older men with MCI who practiced moderate aerobic physical exercise over 6 months had increased BDNF concentrations [32]. In both cases, training protocols were performed three to four times per week, for 45–60 min per session, with intensities between 60–75% of heart rate reserve or estimated maximum heart rate.

On the other hand, studies that analyzed the effect of resistance training on BDNF levels are contradictory. A meta-analysis found that resistance exercise does not seem to change the concentrations of BDNF [33]. However, Pereira et al. [34] evaluated a muscle strength exercise program in elderly patients and found increased peripheral BDNF levels after 10 weeks of training. This protocol consisted of 60-minute sessions, three times per week, with progressive intensities ranging from 60% to 80% of one-repetition maximum (1RM). Multimodal exercise has been shown to be effective at increasing BDNF concentrations in the elderly. Nascimento et al. [35], analyzed the effects of a multimodal exercise program over a 16-week period on peripheral BDNF levels in elderly patients and elderly patients with MCI. The results showed significant improvements in peripheral BDNF levels across all participants. This intervention was conducted three times per week, with 60-minute sessions that integrated aerobic, strength, balance, and cognitive tasks. Another randomized controlled trial evaluated multimodal exercise intervention in a group that attended a 60-minute class twice each week that included motor fitness, cardiovascular exercises, and strength training. This program resulted in increased levels of plasma BDNF in older women when compared with controls [36].

With regard to the cognitive variables evaluated in the study, a significant effect was observed for attention and executive functions (FAB) and executive functions (CDT) after the 12 weeks of multimodal exercise intervention. Results have been conflicting with regard to which cognitive domains are most sensitive to exercise in the elderly. The BAF and CDT were chosen in this study, as both have been used to evaluate executive function in elderly patients with AD, and executive function has been particularly sensitive to the effect of physical exercise in elderly patients with AD [8,37]. The results found in this study are similar to what was found by Pedroso et al. [8], who detected a significant interaction between groups and moments in BAF and a significant difference in CDT between the groups at the time of the intervention, showing the effects of 4 months of multimodal physical exercise on executive function. A study by Coelho et al. [7] investigated the effect of a multimodal exercise intervention program on attention and executive function in elderly patients with AD and found a significant increase in BAF scores in the intervention group when compared to the control group. On the other hand, it was verified that the control group had decreased scores in the CDT. Although between-group comparisons using the Mann–Whitney U-test did not reveal statistically significant differences, the Wilcoxon test demonstrated significant within-group improvements in the intervention group for FAB, CDT, SPPB, and TUG scores. The evaluation of cognitive function related to language was performed using SVFT. The values found were not significant. Although the multimodal exercise intervention encompassed language in cognitive tasks, the training group showed no improvement in language. A study by Öhman et al. [37] also used the same instrument to evaluate language and observed no significant improvement in this parameter. However, some studies that used different instruments to evaluate language and applied programs of physical and cognitive exercises found better performance in the language of elderly patients with AD [6,38].

Some mechanisms associated with physical exercise that contribute to improvement in cognitive functions include neurophysiological benefits, such as increased cerebral blood flow, vascularization, and synthesis of neurotransmitters and enhanced neurotrophic factors (BDNF; IGF-1—insulin-like growth factor-1; vascular endothelial growth factor (VEGF)), which increase neurogenesis, synaptogenesis, and angiogenesis. Increases in BDNF concentrations imply that neurogenesis may be a component of the mechanism underpinning the cognitive improvements associated with multimodal exercise found in this study. Furthermore, in the present study, the executive functions, including mental flexibility, abstraction, programming, and planning, were stimulated during the dual-task portion of the multimodal exercise intervention, which also possibly contributed to the improvement in cognitive function.

The present study also observed motor functionality improvements, particularly in gait, lower limb muscle strength, balance, and mobility. Significant improvements were seen in the total SPPB score and its components (gait speed and strength) following training. In contrast, Pitkälä et al. [39] found functional decline in all groups after long-term exercise, although the deterioration was slower in the intervention groups. Van Uffelen et al. [40] did not observe significant SPPB changes after 6 months of moderate-intensity cycling. However, in the present study, better dynamic balance and mobility were observed based on improved TUG scores. Our multimodal intervention was structured with progressive difficulty in 60-minute sessions three times per week, combining strength, endurance, balance, and dual-task exercises. Such improvements are crucial for daily living activities and may reduce dependency associated with AD.

It is widely accepted that, depending on time and intensity, physical activity influences the immune system in different ways. In general terms, it is known that regular and moderate exercise is helpful, while long-term intensive exercise training may depress the immune system [40]. In our study, we evaluated the concentration of key immune molecules for the functioning of the immune system, the cytokines. We demonstrated that patients with AD have higher IL-2 levels, and the multimodal exercise program resulted in a significant reduction in IL-2 levels, restoring them to levels similar to cognitively healthy individuals. IL-2 is an important cytokine for the process of clonal expansion and maintenance of regulatory T cells [41], and a decrease in this molecule can have profound effects on the generation of the body’s immune response and homeostatic balance. Our results agree with other studies that investigated the role of exercise in modulating the production of this cytokine. The reduction in this molecule can have profound effects on the generation of the body’s immune response and homeostatic balance. Our results are consistent with other studies that have investigated the role of exercise in modulating the production of this cytokine. Malkowska and Sawczuk [42] highlighted that physical activity can modulate the immune response by regulating cytokine production, including IL-2, with effects depending on the intensity and regularity of exercise. As depicted in Figure 3, the other cytokines evaluated in this study were unchanged after multimodal exercise intervention.

Regarding the cytokine levels, the significant reduction in IL-2 observed in the intervention group suggests a potential anti-inflammatory effect of the multimodal exercise program. However, it is important to note that no statistically significant changes were observed in the levels of INF-γ, TNF-α, IL-4, or IL-10. This finding indicates a limited systemic immune response and should be interpreted with caution. Although IL-2 reduction may reflect beneficial immunomodulatory adaptations, the absence of broader cytokine changes suggests that the anti-inflammatory effects may have been modest or localized. These results underscore the need for further studies with larger samples and longer interventions to better elucidate the immunological mechanisms involved.

We suggest, therefore, the adoption of public policies directed towards patients with AD in the mild and moderate stages so that they can benefit from intervention protocols, particularly the protocol developed in this study (multimodal protocol with dual tasks), since physical exercise is an effective non-pharmacological treatment alternative. Thus, those patients may be encouraged to leave their routine activities, stimulating their motor, cognitive, and social living functions to improve performance and immune response in daily life activities. The current intervention could be considered as an add-on therapy in a community setting.

Although the present study included cognitively healthy older adults for comparative analysis of BDNF, IL-2, and cognitive performance, functional and motor parameters were not assessed in this group. Including such outcomes in future trials could enhance the interpretability of motor improvements by offering a broader reference point. Nonetheless, the marked contrast in cognitive scores between AD and healthy controls reinforces the validity of the observed gains within the intervention group. Future studies might build upon this approach to establish functional benchmarks for exercise-induced improvements in older adults with AD.

This study presents some important limitations that should be considered. The small sample size and the absence of randomization limit the statistical power and restrict the generalizability of the findings to other populations. Moreover, this is a preliminary study, and the results should be interpreted with caution. Another relevant limitation is the wide age range of the participants (62 to 85 years), which may have introduced age-related variability, potentially influencing the cognitive and motor performance outcomes. Additionally, the introduction of the cognitive component only in the second half of the intervention may limit the ability to isolate its specific effects. Future studies should consider distinct arms for motor and cognitive–motor interventions. Future research with larger sample sizes, narrower age ranges, and randomized controlled designs is necessary to confirm the observed effects and strengthen the evidence regarding the proposed intervention. Furthermore, as this was a pilot study with multiple outcomes and limited sample size, we treated cognitive, motor, and cytokine outcomes as exploratory. No correction for multiplicity was applied, which increases the possibility of false-positive findings.

## 5. Conclusions

In conclusion, our study demonstrates that a 12-week multimodal exercise intervention, even when partially incorporating cognitive components, can promote relevant cognitive and motor improvements in elderly individuals with AD. These findings reinforce the potential of integrated physical exercise programs as non-pharmacological strategies to support cognitive and functional health in this population.

## Figures and Tables

**Table 1:** Sociodemographic and clinical characteristics of the participants at baseline.

Variables	Intervention Group (*n* = 7)	Control Group (*n* = 8)	*p*
Age (years)	75.7 ± 6.4	76.1 ± 7.4	1
Education (years)	7.6 ± 4.9	8.6 ± 5.7	1
Disease duration (months)	21.6 ± 11.8	40 ± 34.8	0.31
CDR (points)	1.5 ± 0.5	1.6 ± 0.7	0.77
Depressive symptoms (points)	7.5 ± 2.7	6.9 ± 2.3	0.71
MMSE (points)	18.6 ± 4.7	17 ± 7.3	0.65
QBMI (points)	1.8 ± 1.3	2.9 ± 1.7	0.27

Mann–Whitney U-test for independent samples. Age (U = 26.0, *p* = 0.812, r = 0.07), education (U = 24.0, *p* = 0.734, r = 0.10), disease duration (U = 27.0, *p* = 0.845, r = 0.06), CDR (U = 23.0, *p* = 0.701, r = 0.11), depressive symptoms (U = 25.0, *p* = 0.768, r = 0.09), MMSE (U = 22.0, *p* = 0.650, r = 0.12), and QBMI (U = 24.0, *p* = 0.734, r = 0.10).

**Table 2:** Progression of weight training.

Week	1		2	3	4	5	6
Load		2 times, 8 to 10 repetitions		2 times, 10 to 15 repetitions		3 times, 10 to 15 repetitions	
Week	7		8	9	10	11	12
Load		3 times, 10 to 15 repetitions + load increase		3 times, 10 to 15 repetitions + new exercises		3 times, 10 to 15 repetitions + load increase	

**Table 3:** Distribution of variables of cognitive functions and motor functionality pre- and post intervention.

	Intervention Group					Control Group				
Variables	Pre		Post			Pre		Post		
	Mean	SD	Mean	SD	*p* *	Mean	SD	Mean	SD	*p* *
**Cognitive Functions**										
*FAB*	9.36	2.58	10.01	3.85	0.05 *	9.50	4.37	11.63	2.92	0.07
*CDT*	4.00	3.05	5.36	2.90	0.04 *	4.38	3.88	5.13	3.56	0.46
*SVFT*	6.73	4.05	7.36	3.35	0.30	6.13	4.42	7.63	2.85	0.78
**Motor Functionality**										
SPPB (points)	7.36	2.73	9.64	2.65	0.009 *	8.13	3.31	9.13	3.04	0.22
Balance (points)	2.55	0.93	3.18	0.98	0.06	2.50	1.06	3.00	0.92	0.27
Gait (time)	6.57	4.38	3.23	1.33	0.02 *	4.19	2.11	3.95	1.89	0.40
Standing up (time)	17.23	7.32	12.53	4.58	0.008 *	9.57	6.50	10.4	4.48	1.00
TUG (time)	10.92	3.79	8.58	3.60	0.008 *	10.50	4.84	10.61	4.72	1.00
TUG (steps)	14.82	3.51	15.64	4.88	0.32	15.50	3.33	15.51	3.81	0.91
Berg Functional Scale (points)	49.91	4.52	51.36	4.84	0.12	50.25	6.36	52.63	2.77	0.16

* Wilcoxon test; SD = standard deviation; FAB = Frontal Assessment Battery; CDT = Clock Drawing Test; SVFT = Semantic Verbal Fluency Test; SPPB = Short Physical Performance; TUG = Timed Up and Go Test. *p*-values equal to 1.00 result from exact ties in non-parametric tests, indicating no difference between groups.

## Abbreviations

The following abbreviations are used in this manuscript:

ABRAz	Brazilin Association of Alzheimer’s
AD	Alzheimer’s disease
ADI	Alzheimer’s Disease International
BDNF	Brain-derived neurotrophic factor
CDT	Clock Drawing Test
CDR	Clinical Dementia Rating Score
FAB	Frontal Assessment Battery
GDS	Geriatric Depression Scale
MCI	Mild cognitive impairment
MMSE	Mini-Mental State Examination
SPPB	Short Physical Performance Battery
SVFT	Semantic Verbal Fluency Test
UFTM	Federal University of Triângulo Mineiro
UEGF	Vascular endothelial growth factor
